# 9-Benzyl-9*H*-carbazole

**DOI:** 10.1107/S1600536810012444

**Published:** 2010-04-14

**Authors:** Nesimi Uludağ, Murat Ateş, Barış Tercan, Emel Ermiş, Tuncer Hökelek

**Affiliations:** a Namık Kemal University, Faculty of Arts and Sciences, Department of Chemistry, 59100 Tekirdağ, Turkey; b Karabük University, Department of Physics, 78050 Karabük, Turkey; c Anadolu University, Faculty of Science, Department of Chemistry, 26470 Yenibağlar, Eskişehir, Turkey; d Hacettepe University, Department of Physics, 06800 Beytepe, Ankara, Turkey

## Abstract

The asymmetric unit of the title compound, C_19_H_15_N, contains two crystallographically independent mol­ecules. In both mol­ecules, the planar carbazole moieties [maximum deviations = 0.037 (4) and 0.042 (3) Å] are oriented with respect to the adjacent benzene rings, at dihedral angles of 85.29 (8) and 89.89 (7)°, respectively. In the crystal structure, weak C—H⋯π inter­actions are observed involving the carbazole rings.

## Related literature

For tetra­hydro­carbazole systems present in the framework of a number of indole-type alkaloids of biological inter­est, see: Phillipson & Zenk (1980 ▶); Saxton (1983 ▶); Abraham (1975 ▶). For related structures, see: Hökelek *et al.* (1994 ▶, 1998 ▶, 1999 ▶, 2004 ▶, 2006 ▶); Patır *et al.* (1997 ▶); Hökelek & Patır (1999 ▶, 2002 ▶); Çaylak *et al.* (2007 ▶). For bond-length data, see: Allen *et al.* (1987 ▶).
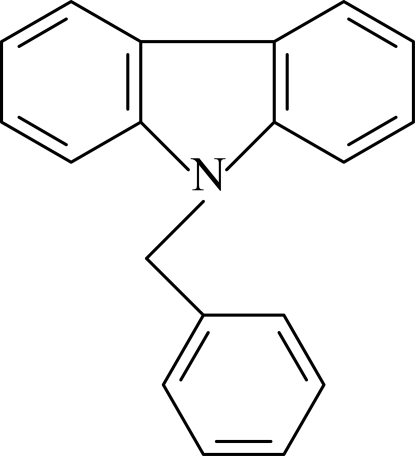


## Experimental

### 

#### Crystal data

C_19_H_15_N
*M* *_r_* = 257.32
Monoclinic, 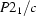

*a* = 14.9305 (4) Å
*b* = 5.5612 (2) Å
*c* = 32.7916 (8) Å
β = 94.518 (3)°
*V* = 2714.27 (14) Å^3^
*Z* = 8
Mo *K*α radiation
μ = 0.07 mm^−1^
*T* = 100 K
0.27 × 0.15 × 0.14 mm

#### Data collection

Bruker Kappa APEXII CCD area-detector diffractometer
Absorption correction: multi-scan (*SADABS*; Bruker, 2005 ▶) *T*_min_ = 0.981, *T*_max_ = 0.990
24870 measured reflections
6816 independent reflections
3384 reflections with *I* > 2σ(*I*)
*R*_int_ = 0.103

#### Refinement

*R*[*F* ^2^ > 2σ(*F* ^2^)] = 0.076
*wR*(*F* ^2^) = 0.210
*S* = 1.03
6816 reflections
474 parameters
H atoms treated by a mixture of independent and constrained refinement
Δρ_max_ = 0.27 e Å^−3^
Δρ_min_ = −0.26 e Å^−3^

### 

Data collection: *APEX2* (Bruker, 2007 ▶); cell refinement: *SAINT* (Bruker, 2007 ▶); data reduction: *SAINT*; program(s) used to solve structure: *SHELXS97* (Sheldrick, 2008 ▶); program(s) used to refine structure: *SHELXL97* (Sheldrick, 2008 ▶); molecular graphics: *ORTEP-3 for Windows* (Farrugia, 1997 ▶); software used to prepare material for publication: *WinGX* (Farrugia, 1999 ▶) and *PLATON* (Spek, 2009 ▶).

## Supplementary Material

Crystal structure: contains datablocks I, global. DOI: 10.1107/S1600536810012444/xu2745sup1.cif

Structure factors: contains datablocks I. DOI: 10.1107/S1600536810012444/xu2745Isup2.hkl

Additional supplementary materials:  crystallographic information; 3D view; checkCIF report

## Figures and Tables

**Table 1:** Hydrogen-bond geometry (Å, °)

*D*—H⋯*A*	*D*—H	H⋯*A*	*D*⋯*A*	*D*—H⋯*A*
C6—H6⋯*Cg*1′^i^	0.97 (4)	2.940 (4)	3.636 (5)	129.46 (5)
C10′—H10*C*⋯*Cg*1′^ii^	0.98 (3)	2.787 (4)	3.700 (5)	154.92 (4)
C4′—H4′⋯*Cg*3^i^	0.99 (4)	2.706 (4)	3.554 (4)	144.36 (5)

Symmetry codes: (i) 
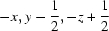
; (ii) 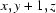. *Cg*1′ and *Cg*3 are the centroids of the C1′–C4′/C4*A*′/C9*A*′ and C5*A*/C5–C8/C8*A* rings, respectively.

**Fig. 1.**
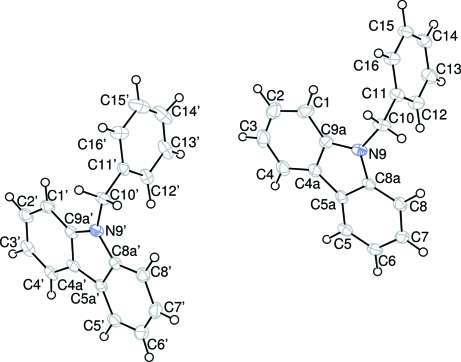
The molecular structure of the title molecule with the atom-numbering scheme. The displacement ellipsoids are drawn at the 50% probability level.

## supplementary crystallographic information

### Comment

Tetrahydrocarbazole systems are present in the framework of a number of indole-type alkaloids of biological interest (Phillipson & Zenk, 1980; Saxton, 1983; Abraham, 1975). The structures of tricyclic, tetracyclic and pentacyclic ring systems with dithiolane and other substituents of the tetrahydrocarbazole core, have been the subject of much interest in our laboratory. These include 1,2,3,4-tetrahydrocarbazole-1-spiro-2'-[1,3]dithiolane, (II) (Hökelek *et al.*, 1994), *N*-(2-methoxyethyl)-*N*-{2,3,4,9-tetrahydrospiro[1*H*-carbazole-1, 2-(1,3)dithiolane]-4-yl}benzene-sulfonamide, (III) (Patır *et al.*, 1997), spiro[carbazole-1(2*H*),2'-[1,3]-dithiolan]-4(3*H*)-one, (IV) (Hökelek *et al.*, 1998), 9-acetonyl-3-ethylidene-1,2,3,4-tetrahydrospiro[carbazole-1,2'-[1,3] dithiolan]-4-one, (V) (Hökelek *et al.*, 1999), *N*-(2,2-dimethoxyethyl)-N -{9-methoxymethyl-1,2,3,4-tetrahydrospiro[carbazole-1,2'-[1,3]dithiolan] -4-yl}benzamide, (VI) (Hökelek & Patır, 1999), 3a,4,10,10 b-tetrahydro-2H -furo[2,3-a]carbazol-5(3*H*)-one, (VII) (Çaylak *et al.*, 2007); also the pentacyclic compounds 6-ethyl-4-(2-methoxyethyl)-2,6-methano-5-oxo-hexahydro- pyrrolo(2,3-d)carbazole-1-spiro-2'-(1,3)dithiolane, (VIII) (Hökelek & Patır, 2002), *N*-(2-benzyloxyethyl)-4,7-dimethyl-6-(1,3-dithiolan-2-yl)-1,2, 3,4,5,6-hexahydro-1,5-methano-2-azocino[4,3-b]indol-2-one, (IX) (Hökelek *et al.*, 2004) and 4-ethyl-6,6-ethylenedithio-2-(2-methoxyethyl)-7-methoxy- methylene-2,3,4,5,6,7-hexahydro-1,5-methano-1*H*-azocino[4,3-b]indol-3-one, (X) (Hökelek *et al.*, 2006). The title compound, (I), may be considered as a synthetic precursor of tetracyclic indole alkaloids of biological interests. The present study was undertaken to ascertain its crystal structure.

The title compound consists of a carbazole skeleton with a benzyl group. Its asymmetric unit, (Fig. 1), contains two crystallographically independent molecules, where the bond lengths (Allen *et al.*, 1987) and angles are within normal ranges, and generally agree with those in compounds (II)-(X). In all structures atom N9 is substituted.

An examination of the deviations from the least-squares planes through individual rings shows that rings A (C1—C4/C4a/C9a), B (C4a/C5a/C8a/N9/C9a), C (C5a/C5—C8/C8a), D (C11—C16) and A' (C1'-C4'/C4a'/C9a'), B' (C4a'/C5a'/ C8a'/N9'/C9a'), C' (C5a'/C5'-C8'/C8a'), D' (C11'-C16') are planar. The carbazole skeletons, containing the rings A, B, C and A', B', C' are also nearly coplanar [with a maximum deviations of 0.037 (4) and 0.042 (3) Å for atoms C2 and C7', respectively] with dihedral angles of A/B = 1.28 (10), A/C = 1.57 (9), B/C = 0.32 (7) ° and A'/B' = 0.94 (10), A'/C' = 2.37 (10), B'/C' = 1.72 (11) °. Rings D and D' are oriented with respect to the planar carbazole skeletons at dihedral angles of 85.29 (8) and 89.89 (7) °, respectively. Atoms C10 and C10' displaced by -0.109 (3), -0.005 (4) Å and -0.016 (3), -0.098 (3) Å from the planes of the corresponding carbazole skeletons and benzene rings, respectively.

In the crystal structure, three weak C—H···π interactions (Table 1) involving the carbazole rings are observed.

### Experimental

For the preparation of the title compound, (I), sodium hydride (2.38 g, 59.85 mmol) was added to a solution of carbazole (5.00 g, 29.92 mmol) in dry tetrahydrofuran (200 ml) in several portions, and stirred at room temperature for 1 h under argon atmosphere. Then, benzylchloride (5.68 g, 44.88 mmol) was added and stirred at 343 K for 6 h. The reaction mixture was cooled in an ice bath, and hydrochloric acid (10%, 200 ml) was added. After the extraction with dichloromethane (300 ml), the organic layer was dried over anhydrous magnesium sulfate and the solvent was evaporated under reduced pressure. The residue was purified by column chromatograpy using silica gel and dichloromethane-petroleum ether (1:1), and the product was recrystallized from diethyl ether and cyclohexane mixture (1:1) (yield; 4.00 g, 80%, m.p. 388 K).

### Refinement

H3 and H7' atoms were positioned geometrically, with C—H = 0.93 Å for aromatic H atoms and constrained to ride on their parent atoms, with U_iso_(H) = 1.2U_eq_(C). The remaining H atoms were located in difference synthesis and refined isotropically.

### Crystal data

**Table:**

C_19_H_15_N	*F*(000) = 1088
*M**_r_* = 257.32	*D*_x_ = 1.259 Mg m^−^^3^
Monoclinic, *P*2_1_/*c*	Mo *K*α radiation, λ = 0.71073 Å
Hall symbol: -P 2ybc	Cell parameters from 1669 reflections
*a* = 14.9305 (4) Å	θ = 2.5–22.9°
*b* = 5.5612 (2) Å	µ = 0.07 mm^−^^1^
*c* = 32.7916 (8) Å	*T* = 100 K
β = 94.518 (3)°	Block, colorless
*V* = 2714.27 (14) Å^3^	0.27 × 0.15 × 0.14 mm
*Z* = 8	

### Data collection

**Table:**

Bruker Kappa APEXII CCD area-detector diffractometer	6816 independent reflections
Radiation source: fine-focus sealed tube	3384 reflections with *I* > 2σ(*I*)
graphite	*R*_int_ = 0.103
φ and ω scans	θ_max_ = 28.4°, θ_min_ = 1.3°
Absorption correction: multi-scan (*SADABS*; Bruker, 2005)	*h* = −19→18
*T*_min_ = 0.981, *T*_max_ = 0.990	*k* = −7→7
24870 measured reflections	*l* = −42→43

### Refinement

**Table:**

Refinement on *F*^2^	Secondary atom site location: difference Fourier map
Least-squares matrix: full	Hydrogen site location: inferred from neighbouring sites
*R*[*F*^2^ > 2σ(*F*^2^)] = 0.076	H atoms treated by a mixture of independent and constrained refinement
*wR*(*F*^2^) = 0.210	*w* = 1/[σ^2^(*F*_o_^2^) + (0.0918*P*)^2^] where *P* = (*F*_o_^2^ + 2*F*_c_^2^)/3
*S* = 1.03	(Δ/σ)_max_ < 0.001
6816 reflections	Δρ_max_ = 0.27 e Å^−^^3^
474 parameters	Δρ_min_ = −0.26 e Å^−^^3^
0 restraints	Extinction correction: *SHELXL97* (Sheldrick, 2008), Fc^*^=kFc[1+0.001xFc^2^λ^3^/sin(2θ)]^-1/4^
Primary atom site location: structure-invariant direct methods	Extinction coefficient: 0.0068 (11)

### Special details

**Table:**

Geometry. All esds (except the esd in the dihedral angle between two l.s. planes) are estimated using the full covariance matrix. The cell esds are taken into account individually in the estimation of esds in distances, angles and torsion angles; correlations between esds in cell parameters are only used when they are defined by crystal symmetry. An approximate (isotropic) treatment of cell esds is used for estimating esds involving l.s. planes.
Refinement. Refinement of *F*^2^ against ALL reflections. The weighted *R*-factor wR and goodness of fit *S* are based on *F*^2^, conventional *R*-factors *R* are based on *F*, with *F* set to zero for negative *F*^2^. The threshold expression of *F*^2^ > σ(*F*^2^) is used only for calculating *R*-factors(gt) etc. and is not relevant to the choice of reflections for refinement. *R*-factors based on *F*^2^ are statistically about twice as large as those based on *F*, and *R*- factors based on ALL data will be even larger.

### Fractional atomic coordinates and isotropic or equivalent isotropic displacement parameters (Å^2^)

**Table:**

	*x*	*y*	*z*	*U*_iso_*/*U*_eq_	
C1	0.5696 (2)	0.6160 (7)	0.83884 (11)	0.0399 (9)	
H1	0.536 (2)	0.451 (6)	0.8446 (9)	0.042 (9)*	
C2	0.5654 (2)	0.7192 (8)	0.80036 (11)	0.0495 (10)	
H2	0.524 (2)	0.643 (6)	0.7769 (10)	0.040 (9)*	
C3	0.6133 (2)	0.9241 (8)	0.79216 (10)	0.0486 (10)	
H3	0.6077	0.9890	0.7659	0.058*	
C4	0.6692 (2)	1.0341 (7)	0.82209 (10)	0.0364 (8)	
H4	0.702 (2)	1.169 (6)	0.8155 (9)	0.030 (9)*	
C4A	0.67613 (19)	0.9336 (6)	0.86118 (9)	0.0286 (7)	
C5	0.7898 (2)	1.1740 (6)	0.91002 (10)	0.0286 (7)	
H5	0.807 (2)	1.303 (6)	0.8898 (10)	0.052 (10)*	
C5A	0.72814 (19)	0.9919 (5)	0.89881 (8)	0.0244 (6)	
C6	0.8284 (2)	1.1795 (6)	0.94938 (10)	0.0308 (7)	
H6	0.870 (2)	1.308 (6)	0.9577 (9)	0.045 (10)*	
C7	0.8064 (2)	1.0064 (6)	0.97798 (10)	0.0294 (7)	
H7	0.836 (2)	1.010 (6)	1.0055 (10)	0.037 (9)*	
C8	0.7450 (2)	0.8268 (6)	0.96804 (9)	0.0284 (7)	
H8	0.727 (2)	0.696 (6)	0.9883 (10)	0.045 (9)*	
C8A	0.70640 (19)	0.8208 (5)	0.92781 (9)	0.0243 (6)	
N9	0.64413 (16)	0.6597 (4)	0.90964 (7)	0.0263 (6)	
C9A	0.62622 (19)	0.7261 (5)	0.86924 (9)	0.0274 (7)	
C10	0.5991 (2)	0.4654 (6)	0.92981 (11)	0.0314 (7)	
H10A	0.604 (2)	0.313 (6)	0.9142 (9)	0.036 (9)*	
H10B	0.632 (2)	0.433 (5)	0.9553 (9)	0.031 (9)*	
C11	0.50251 (19)	0.5224 (5)	0.93644 (9)	0.0260 (7)	
C12	0.4798 (2)	0.7304 (6)	0.95693 (10)	0.0335 (8)	
H12	0.527 (2)	0.852 (6)	0.9672 (10)	0.048 (10)*	
C13	0.3908 (2)	0.7795 (6)	0.96297 (10)	0.0348 (8)	
H13	0.375 (2)	0.931 (6)	0.9762 (10)	0.052 (10)*	
C14	0.3239 (2)	0.6231 (6)	0.94863 (9)	0.0311 (7)	
H14	0.263 (3)	0.675 (6)	0.9533 (10)	0.054 (11)*	
C15	0.3458 (2)	0.4166 (6)	0.92833 (10)	0.0337 (8)	
H15	0.296 (2)	0.295 (6)	0.9180 (9)	0.038 (9)*	
C16	0.4348 (2)	0.3666 (6)	0.92232 (10)	0.0305 (7)	
H16	0.4532 (19)	0.217 (5)	0.9088 (8)	0.027 (8)*	
C1'	0.9099 (2)	0.8115 (6)	0.57706 (9)	0.0291 (7)	
H1'	0.865 (2)	0.673 (5)	0.5719 (8)	0.032 (8)*	
C2'	0.9229 (2)	0.9787 (6)	0.54711 (10)	0.0333 (8)	
H2'	0.883 (2)	0.951 (5)	0.5217 (9)	0.029 (8)*	
C3'	0.9879 (2)	1.1600 (6)	0.55305 (10)	0.0345 (8)	
H3'	0.998 (2)	1.271 (6)	0.5318 (9)	0.035 (9)*	
C4'	1.0415 (2)	1.1763 (6)	0.58923 (10)	0.0294 (7)	
H4'	1.085 (3)	1.310 (7)	0.5918 (11)	0.063 (12)*	
C4A'	1.02988 (19)	1.0096 (5)	0.61992 (9)	0.0258 (7)	
C5'	1.1437 (2)	1.0841 (6)	0.68366 (10)	0.0325 (8)	
H5'	1.177 (2)	1.222 (6)	0.6724 (9)	0.041 (9)*	
C5A'	1.07331 (19)	0.9688 (5)	0.66042 (9)	0.0258 (7)	
C6'	1.1713 (2)	0.9928 (7)	0.72151 (10)	0.0399 (8)	
H6'	1.223 (3)	1.067 (7)	0.7361 (11)	0.061 (11)*	
C7'	1.1302 (2)	0.7899 (6)	0.73662 (9)	0.0374 (8)	
H7'	1.1509	0.7306	0.7622	0.045*	
C8'	1.0594 (2)	0.6735 (6)	0.71486 (9)	0.0305 (7)	
H8'	1.028 (2)	0.519 (6)	0.7270 (9)	0.043 (9)*	
C8A'	1.03215 (19)	0.7642 (5)	0.67661 (8)	0.0249 (7)	
N9'	0.96646 (16)	0.6806 (4)	0.64747 (7)	0.0255 (6)	
C9A'	0.96460 (19)	0.8272 (5)	0.61347 (9)	0.0258 (7)	
C10'	0.9071 (2)	0.4776 (5)	0.65181 (10)	0.0267 (7)	
H10C	0.910 (2)	0.373 (6)	0.6277 (10)	0.042 (10)*	
H10D	0.932 (2)	0.372 (5)	0.6747 (9)	0.033 (9)*	
C11'	0.81229 (19)	0.5454 (5)	0.66032 (8)	0.0241 (6)	
C12'	0.7931 (2)	0.7589 (6)	0.67972 (9)	0.0314 (7)	
H12'	0.847 (2)	0.884 (6)	0.6895 (9)	0.047 (10)*	
C13'	0.7052 (2)	0.8047 (7)	0.68913 (11)	0.0401 (9)	
H13'	0.690 (2)	0.951 (6)	0.7018 (9)	0.042 (10)*	
C14'	0.6376 (2)	0.6429 (7)	0.67937 (12)	0.0481 (10)	
H14'	0.576 (3)	0.680 (7)	0.6901 (12)	0.075 (13)*	
C15'	0.6564 (2)	0.4339 (7)	0.65933 (12)	0.0462 (10)	
H15'	0.605 (3)	0.327 (6)	0.6506 (10)	0.054 (11)*	
C16'	0.7434 (2)	0.3849 (6)	0.64961 (10)	0.0350 (8)	
H16'	0.758 (2)	0.242 (6)	0.6364 (10)	0.041 (10)*	

### Atomic displacement parameters (Å^2^)

**Table:**

	*U*^11^	*U*^22^	*U*^33^	*U*^12^	*U*^13^	*U*^23^
C1	0.0220 (17)	0.053 (2)	0.045 (2)	−0.0002 (16)	0.0017 (14)	−0.0173 (18)
C2	0.029 (2)	0.085 (3)	0.034 (2)	0.009 (2)	−0.0012 (16)	−0.015 (2)
C3	0.031 (2)	0.086 (3)	0.0284 (18)	0.015 (2)	0.0033 (15)	0.0019 (19)
C4	0.0239 (18)	0.055 (2)	0.0316 (19)	0.0087 (17)	0.0072 (14)	0.0043 (17)
C4A	0.0183 (15)	0.0393 (18)	0.0288 (17)	0.0061 (14)	0.0056 (12)	−0.0019 (14)
C5	0.0219 (16)	0.0278 (16)	0.0372 (19)	0.0003 (13)	0.0090 (13)	−0.0011 (14)
C5A	0.0196 (15)	0.0290 (15)	0.0254 (15)	0.0057 (13)	0.0075 (12)	0.0015 (13)
C6	0.0225 (17)	0.0326 (17)	0.0379 (19)	−0.0054 (14)	0.0062 (14)	−0.0049 (15)
C7	0.0226 (16)	0.0366 (17)	0.0291 (17)	−0.0006 (14)	0.0024 (13)	−0.0058 (15)
C8	0.0247 (17)	0.0331 (16)	0.0280 (17)	0.0018 (14)	0.0059 (13)	0.0017 (14)
C8A	0.0172 (15)	0.0259 (15)	0.0308 (16)	0.0018 (12)	0.0074 (12)	−0.0026 (13)
N9	0.0188 (13)	0.0243 (12)	0.0358 (15)	−0.0004 (10)	0.0034 (10)	−0.0009 (11)
C9A	0.0153 (15)	0.0365 (17)	0.0305 (17)	0.0066 (13)	0.0030 (12)	−0.0057 (14)
C10	0.0203 (16)	0.0262 (17)	0.048 (2)	−0.0022 (13)	0.0043 (15)	0.0017 (16)
C11	0.0223 (16)	0.0238 (14)	0.0321 (16)	0.0021 (13)	0.0025 (12)	0.0043 (13)
C12	0.0273 (18)	0.0325 (17)	0.0408 (19)	−0.0022 (15)	0.0035 (14)	−0.0034 (15)
C13	0.0306 (19)	0.0331 (18)	0.042 (2)	0.0030 (15)	0.0097 (14)	−0.0013 (16)
C14	0.0210 (17)	0.0367 (18)	0.0360 (18)	0.0040 (15)	0.0046 (13)	0.0088 (15)
C15	0.0236 (17)	0.0416 (19)	0.0357 (18)	−0.0096 (15)	0.0011 (14)	0.0044 (16)
C16	0.0252 (17)	0.0284 (17)	0.0378 (19)	−0.0031 (14)	0.0021 (13)	−0.0021 (14)
C1'	0.0205 (16)	0.0371 (18)	0.0298 (17)	0.0043 (14)	0.0024 (13)	0.0036 (15)
C2'	0.0202 (16)	0.048 (2)	0.0318 (18)	0.0100 (15)	0.0054 (13)	0.0058 (16)
C3'	0.0312 (19)	0.0403 (19)	0.0336 (19)	0.0107 (15)	0.0126 (15)	0.0119 (16)
C4'	0.0245 (17)	0.0282 (16)	0.0376 (19)	0.0042 (14)	0.0154 (14)	0.0013 (14)
C4A'	0.0193 (15)	0.0261 (15)	0.0333 (16)	0.0024 (13)	0.0099 (12)	−0.0013 (13)
C5'	0.0248 (17)	0.0365 (18)	0.0378 (19)	−0.0033 (15)	0.0115 (14)	−0.0101 (15)
C5A'	0.0202 (15)	0.0294 (15)	0.0287 (16)	0.0016 (13)	0.0081 (12)	−0.0049 (13)
C6'	0.0304 (19)	0.054 (2)	0.0357 (19)	−0.0029 (18)	0.0048 (15)	−0.0164 (18)
C7'	0.0321 (19)	0.055 (2)	0.0249 (17)	0.0100 (17)	0.0017 (13)	−0.0072 (16)
C8'	0.0268 (17)	0.0378 (18)	0.0274 (17)	0.0045 (14)	0.0042 (13)	0.0007 (15)
C8A'	0.0199 (15)	0.0291 (16)	0.0263 (16)	0.0024 (13)	0.0060 (12)	−0.0036 (13)
N9'	0.0202 (13)	0.0284 (13)	0.0280 (13)	−0.0010 (11)	0.0030 (10)	0.0035 (11)
C9A'	0.0197 (15)	0.0291 (15)	0.0293 (16)	0.0026 (13)	0.0070 (12)	0.0011 (13)
C10'	0.0214 (16)	0.0252 (15)	0.0344 (18)	−0.0027 (13)	0.0075 (13)	−0.0020 (15)
C11'	0.0229 (15)	0.0278 (15)	0.0219 (15)	0.0012 (13)	0.0041 (12)	0.0057 (13)
C12'	0.0312 (18)	0.0352 (18)	0.0288 (17)	0.0031 (15)	0.0092 (13)	0.0028 (14)
C13'	0.039 (2)	0.043 (2)	0.042 (2)	0.0130 (18)	0.0205 (16)	0.0135 (18)
C14'	0.027 (2)	0.063 (3)	0.056 (2)	0.0101 (19)	0.0134 (17)	0.031 (2)
C15'	0.0245 (19)	0.051 (2)	0.062 (2)	−0.0046 (18)	−0.0030 (17)	0.022 (2)
C16'	0.0276 (18)	0.0325 (18)	0.044 (2)	−0.0049 (15)	−0.0042 (15)	0.0083 (16)

### Geometric parameters (Å, °)

**Table:**

C1—C2	1.383 (5)	C1'—C2'	1.377 (4)
C1—H1	1.07 (3)	C1'—H1'	1.03 (3)
C2—H2	1.04 (3)	C2'—H2'	0.99 (3)
C3—C2	1.383 (6)	C3'—C2'	1.403 (5)
C3—H3	0.9300	C3'—H3'	0.95 (3)
C4—C3	1.380 (5)	C4'—C3'	1.381 (5)
C4—H4	0.93 (3)	C4'—H4'	0.99 (4)
C4A—C4	1.395 (4)	C4A'—C4'	1.389 (4)
C5—H5	1.02 (4)	C4A'—C5A'	1.449 (4)
C5A—C4A	1.442 (4)	C5'—C6'	1.374 (5)
C5A—C5	1.398 (4)	C5'—H5'	1.00 (3)
C6—C5	1.372 (4)	C5A'—C5'	1.404 (4)
C6—C7	1.401 (4)	C6'—H6'	0.97 (4)
C6—H6	0.97 (4)	C7'—C6'	1.394 (5)
C7—H7	0.97 (3)	C7'—H7'	0.9300
C8—C7	1.377 (4)	C8'—C7'	1.387 (4)
C8—H8	1.04 (3)	C8'—H8'	1.07 (3)
C8A—C5A	1.401 (4)	C8A'—C5A'	1.416 (4)
C8A—C8	1.398 (4)	C8A'—C8'	1.383 (4)
N9—C8A	1.391 (4)	N9'—C8A'	1.394 (4)
N9—C9A	1.381 (4)	N9'—C9A'	1.379 (4)
N9—C10	1.458 (4)	N9'—C10'	1.450 (4)
C9A—C1	1.396 (4)	C9A'—C1'	1.395 (4)
C9A—C4A	1.410 (4)	C9A'—C4A'	1.411 (4)
C10—C11	1.509 (4)	C10'—C11'	1.511 (4)
C10—H10A	1.00 (3)	C10'—H10C	0.98 (3)
C10—H10B	0.95 (3)	C10'—H10D	1.00 (3)
C11—C12	1.393 (4)	C11'—C12'	1.388 (4)
C11—C16	1.384 (4)	C11'—C16'	1.386 (4)
C12—H12	1.02 (4)	C12'—C13'	1.395 (4)
C13—C12	1.386 (4)	C12'—H12'	1.09 (3)
C13—H13	0.98 (3)	C13'—H13'	0.95 (3)
C14—C13	1.379 (5)	C14'—C13'	1.371 (5)
C14—C15	1.379 (5)	C14'—C15'	1.375 (6)
C14—H14	0.99 (4)	C14'—H14'	1.03 (4)
C15—C16	1.387 (4)	C15'—H15'	1.00 (4)
C15—H15	1.04 (3)	C16'—C15'	1.388 (5)
C16—H16	0.99 (3)	C16'—H16'	0.94 (3)
			
C2—C1—C9A	116.8 (4)	C2'—C1'—C9A'	117.5 (3)
C2—C1—H1	122.0 (17)	C2'—C1'—H1'	121.1 (16)
C9A—C1—H1	121.0 (17)	C9A'—C1'—H1'	121.2 (16)
C1—C2—H2	119.3 (18)	C1'—C2'—C3'	121.4 (3)
C3—C2—C1	122.1 (3)	C1'—C2'—H2'	112.6 (17)
C3—C2—H2	118.6 (18)	C3'—C2'—H2'	126.0 (17)
C2—C3—H3	119.3	C2'—C3'—H3'	120.5 (19)
C4—C3—C2	121.3 (3)	C4'—C3'—C2'	121.0 (3)
C4—C3—H3	119.3	C4'—C3'—H3'	118.5 (19)
C3—C4—C4A	118.2 (4)	C3'—C4'—C4A'	118.8 (3)
C3—C4—H4	119.5 (19)	C3'—C4'—H4'	117 (2)
C4A—C4—H4	122.2 (19)	C4A'—C4'—H4'	124 (2)
C4—C4A—C9A	119.9 (3)	C4'—C4A'—C5A'	134.1 (3)
C4—C4A—C5A	133.8 (3)	C4'—C4A'—C9A'	119.7 (3)
C9A—C4A—C5A	106.3 (3)	C9A'—C4A'—C5A'	106.2 (2)
C5A—C5—H5	122 (2)	C5A'—C5'—H5'	121.5 (18)
C6—C5—C5A	118.8 (3)	C6'—C5'—C5A'	118.7 (3)
C6—C5—H5	119 (2)	C6'—C5'—H5'	119.6 (19)
C5—C5A—C4A	133.4 (3)	C5'—C5A'—C4A'	133.5 (3)
C5—C5A—C8A	119.8 (3)	C5'—C5A'—C8A'	119.5 (3)
C8A—C5A—C4A	106.8 (3)	C8A'—C5A'—C4A'	107.1 (2)
C5—C6—C7	120.7 (3)	C5'—C6'—C7'	120.7 (3)
C5—C6—H6	119.5 (19)	C5'—C6'—H6'	117 (2)
C7—C6—H6	119.8 (19)	C7'—C6'—H6'	122 (2)
C6—C7—H7	120 (2)	C6'—C7'—H7'	118.9
C8—C7—C6	121.8 (3)	C8'—C7'—C6'	122.3 (3)
C8—C7—H7	118.5 (19)	C8'—C7'—H7'	118.9
C7—C8—C8A	117.2 (3)	C7'—C8'—H8'	121.4 (17)
C7—C8—H8	124.1 (18)	C8A'—C8'—C7'	117.0 (3)
C8A—C8—H8	118.6 (19)	C8A'—C8'—H8'	121.6 (17)
N9—C8A—C5A	109.2 (2)	N9'—C8A'—C5A'	108.3 (2)
N9—C8A—C8	129.2 (3)	C8'—C8A'—C5A'	121.8 (3)
C8—C8A—C5A	121.6 (3)	C8'—C8A'—N9'	129.9 (3)
C8A—N9—C10	126.8 (3)	C8A'—N9'—C10'	126.5 (2)
C9A—N9—C8A	108.1 (2)	C9A'—N9'—C8A'	109.0 (2)
C9A—N9—C10	124.9 (3)	C9A'—N9'—C10'	124.5 (2)
N9—C9A—C1	128.9 (3)	C1'—C9A'—C4A'	121.6 (3)
N9—C9A—C4A	109.5 (3)	N9'—C9A'—C1'	128.9 (3)
C1—C9A—C4A	121.6 (3)	N9'—C9A'—C4A'	109.5 (2)
N9—C10—C11	112.9 (2)	N9'—C10'—C11'	114.4 (2)
N9—C10—H10B	108.5 (19)	N9'—C10'—H10C	108.6 (19)
N9—C10—H10A	109.9 (18)	N9'—C10'—H10D	109.6 (18)
C11—C10—H10B	110.0 (18)	C11'—C10'—H10C	113.1 (19)
C11—C10—H10A	111.5 (18)	C11'—C10'—H10D	108.0 (18)
H10B—C10—H10A	104 (3)	H10C—C10'—H10D	103 (2)
C12—C11—C10	121.0 (3)	C12'—C11'—C10'	121.9 (3)
C16—C11—C10	120.1 (3)	C16'—C11'—C10'	118.7 (3)
C16—C11—C12	118.9 (3)	C16'—C11'—C12'	119.4 (3)
C11—C12—H12	121.7 (19)	C11'—C12'—C13'	119.3 (3)
C13—C12—C11	120.3 (3)	C11'—C12'—H12'	120.4 (18)
C13—C12—H12	118.0 (19)	C13'—C12'—H12'	120.1 (18)
C12—C13—H13	120 (2)	C12'—C13'—H13'	121 (2)
C14—C13—C12	120.3 (3)	C14'—C13'—C12'	121.1 (4)
C14—C13—H13	120 (2)	C14'—C13'—H13'	118 (2)
C13—C14—C15	119.8 (3)	C13'—C14'—C15'	119.4 (3)
C13—C14—H14	115 (2)	C13'—C14'—H14'	117 (2)
C15—C14—H14	125 (2)	C15'—C14'—H14'	124 (2)
C14—C15—C16	120.1 (3)	C14'—C15'—C16'	120.4 (4)
C14—C15—H15	120.5 (18)	C14'—C15'—H15'	117 (2)
C16—C15—H15	119.3 (18)	C16'—C15'—H15'	122 (2)
C11—C16—C15	120.7 (3)	C11'—C16'—C15'	120.3 (3)
C11—C16—H16	117.0 (17)	C11'—C16'—H16'	118 (2)
C15—C16—H16	122.3 (18)	C15'—C16'—H16'	122 (2)
			
C9A—C1—C2—C3	−1.1 (5)	C9A'—C1'—C2'—C3'	−0.8 (5)
C4—C3—C2—C1	1.0 (6)	C4'—C3'—C2'—C1'	0.3 (5)
C4A—C4—C3—C2	−0.2 (5)	C4A'—C4'—C3'—C2'	−0.2 (4)
C5A—C4A—C4—C3	177.8 (3)	C5A'—C4A'—C4'—C3'	178.8 (3)
C9A—C4A—C4—C3	−0.5 (5)	C9A'—C4A'—C4'—C3'	0.5 (4)
C5—C5A—C4A—C4	1.2 (6)	C4'—C4A'—C5A'—C5'	0.0 (6)
C5—C5A—C4A—C9A	179.7 (3)	C4'—C4A'—C5A'—C8A'	−178.6 (3)
C8A—C5A—C4A—C4	−179.4 (3)	C9A'—C4A'—C5A'—C5'	178.5 (3)
C8A—C5A—C4A—C9A	−0.9 (3)	C9A'—C4A'—C5A'—C8A'	−0.2 (3)
C8A—C5A—C5—C6	0.6 (4)	C5A'—C5'—C6'—C7'	−0.1 (5)
C4A—C5A—C5—C6	179.9 (3)	C4A'—C5A'—C5'—C6'	−177.8 (3)
C7—C6—C5—C5A	−0.2 (4)	C8A'—C5A'—C5'—C6'	0.7 (4)
C5—C6—C7—C8	−0.7 (5)	C8'—C7'—C6'—C5'	−1.0 (5)
C8A—C8—C7—C6	1.2 (4)	C8A'—C8'—C7'—C6'	1.5 (5)
C8—C8A—C5A—C4A	−179.5 (3)	N9'—C8A'—C5A'—C4A'	0.3 (3)
C8—C8A—C5A—C5	0.0 (4)	N9'—C8A'—C5A'—C5'	−178.5 (3)
N9—C8A—C5A—C4A	0.4 (3)	C8'—C8A'—C5A'—C4A'	178.7 (3)
N9—C8A—C5A—C5	179.9 (2)	C8'—C8A'—C5A'—C5'	−0.1 (4)
N9—C8A—C8—C7	179.2 (3)	N9'—C8A'—C8'—C7'	177.1 (3)
C5A—C8A—C8—C7	−0.8 (4)	C5A'—C8A'—C8'—C7'	−0.9 (4)
C9A—N9—C8A—C5A	0.2 (3)	C9A'—N9'—C8A'—C5A'	−0.3 (3)
C9A—N9—C8A—C8	−179.8 (3)	C9A'—N9'—C8A'—C8'	−178.6 (3)
C10—N9—C8A—C5A	−175.1 (3)	C10'—N9'—C8A'—C8'	3.0 (5)
C10—N9—C8A—C8	4.8 (5)	C10'—N9'—C8A'—C5A'	−178.7 (3)
C8A—N9—C9A—C1	178.6 (3)	C8A'—N9'—C9A'—C1'	179.9 (3)
C8A—N9—C9A—C4A	−0.8 (3)	C8A'—N9'—C9A'—C4A'	0.2 (3)
C10—N9—C9A—C1	−5.9 (5)	C10'—N9'—C9A'—C1'	−1.7 (5)
C10—N9—C9A—C4A	174.7 (3)	C10'—N9'—C9A'—C4A'	178.6 (3)
C8A—N9—C10—C11	104.4 (3)	C8A'—N9'—C10'—C11'	102.9 (3)
C9A—N9—C10—C11	−70.2 (4)	C9A'—N9'—C10'—C11'	−75.2 (4)
N9—C9A—C1—C2	−178.9 (3)	N9'—C9A'—C1'—C2'	−178.5 (3)
C4A—C9A—C1—C2	0.5 (5)	C4A'—C9A'—C1'—C2'	1.1 (4)
N9—C9A—C4A—C4	179.8 (3)	N9'—C9A'—C4A'—C4'	178.7 (2)
N9—C9A—C4A—C5A	1.1 (3)	N9'—C9A'—C4A'—C5A'	0.0 (3)
C1—C9A—C4A—C4	0.3 (4)	C1'—C9A'—C4A'—C4'	−1.0 (4)
C1—C9A—C4A—C5A	−178.4 (3)	C1'—C9A'—C4A'—C5A'	−179.7 (3)
N9—C10—C11—C12	−54.4 (4)	N9'—C10'—C11'—C16'	155.3 (3)
N9—C10—C11—C16	125.9 (3)	N9'—C10'—C11'—C12'	−26.9 (4)
C10—C11—C12—C13	−179.6 (3)	C10'—C11'—C12'—C13'	−175.9 (3)
C16—C11—C12—C13	0.0 (5)	C16'—C11'—C12'—C13'	1.9 (4)
C10—C11—C16—C15	179.8 (3)	C10'—C11'—C16'—C15'	175.8 (3)
C12—C11—C16—C15	0.2 (5)	C12'—C11'—C16'—C15'	−2.0 (5)
C14—C13—C12—C11	−0.2 (5)	C11'—C12'—C13'—C14'	−0.3 (5)
C15—C14—C13—C12	0.2 (5)	C15'—C14'—C13'—C12'	−1.1 (5)
C13—C14—C15—C16	−0.1 (5)	C13'—C14'—C15'—C16'	1.0 (5)
C14—C15—C16—C11	−0.1 (5)	C11'—C16'—C15'—C14'	0.5 (5)

### Hydrogen-bond geometry (Å, °)

**Table:**

*D*—H···*A*	*D*—H	H···*A*	*D*···*A*	*D*—H···*A*
C6—H6···Cg1'^i^	0.97 (4)	2.940 (4)	3.636 (5)	129.46 (5)
C10'—H10C···Cg1'^ii^	0.98 (3)	2.787 (4)	3.700 (5)	154.92 (4)
C4'—H4'···Cg3^i^	0.99 (4)	2.706 (4)	3.554 (4)	144.36 (5)

Symmetry codes: (i) −*x*, *y*−1/2, −*z*+1/2; (ii) *x*, *y*+1, *z*.
